# Smoking, Alcohol, Sleep and Risk of Idiopathic Sudden Deafness: A Case-Control Study Using Pooled Controls

**DOI:** 10.2188/jea.11.81

**Published:** 2007-11-30

**Authors:** Mieko Nakamura, Nobuo Aoki, Tsutomu Nakashima, Tomoyuki Hoshino, Tetsuji Yokoyama, Seiji Morioka, Takashi Kawamura, Heizo Tanaka, Tsutomu Hashimoto, Yoshiyuki Ohno, Gary Whitlock

**Affiliations:** 1 Department of Hygiene, Hamamatsu University School of Medicine.; 2 Clinical Trials Research Unit, University of Auckland, New Zealand.; 3 Department of Otorhinolaryngology, Nagoya University School of Medicine.; 4 Department of Otorhinolaryngology, Hamamatsu University School of Medicine.; 5 Department of Epidemiology, Medical Research Institute, Tokyo Medical and Dental University.; 6 Koza Public Health Centre.; 7 Kyoto University Health Service.; 8 Department of Public Health, Wakayama Medical College.; 9 Department of Preventive Medicine, Nagoya University School of Medicine.

**Keywords:** sudden deafness, case-control study, smoking, alcohol, sleep

## Abstract

Sudden deafness sometimes has an identifiable cause, but in most cases the cause is unknown (idiopathic sudden deafness). Vascular impairment has been proposed as an aetiological mechanism for this condition, but it is unclear whether traditional cardiovascular risk factors, such as smoking or alcohol intake, are associated with this condition. We accordingly investigated associations of idiopathic sudden deafness with smoking, alcohol intake and sleep duration in a case-control study. Cases were consecutive patients diagnosed with idiopathic sudden deafness between October 1996 and August 1998 at collaborating hospitals in Japan. Controls were obtained from a nationwide database of pooled controls, with matching for age, gender and residential district. Exposure variables were assessed from a self-administered questionnaire. Subgroup analyses were performed using audiometric subtypes of sudden deafness.

Data were obtained for 164 cases and 20,313 controls. Increased risks of idiopathic sudden deafness were observed among participants who consumed two or more units of alcohol per day (OR=1.90, 95% CI=1.12-3.21), and among participants who slept less than seven hours per night (OR=1.61, 95% CI=1.09-2.37). The direct association with alcohol intake was particularly strong for the participants with profound hearing loss. There was little evidence of an association with smoking. This study suggests that alcohol intake and short sleep duration might be risk factors for idiopathic sudden deafness.

## INTRODUCTION

Sudden deafness can be a distressing experience for patients because it typically has an abrupt onset, and because it is sometimes severe and refractory to treatment^1^^)^. More than four-fifths of cases of sudden deafness do not have an identifiable cause (so-called “idiopathic” sudden deafness^2^^)^). A number of hypothetical causes have been proposed for idiopathic sudden deafness, of which the most plausible are vascular impairment and viral infection^1^^)^. The vascular hypothesis is plausible because the cochlea is a highly vascularized organ supplied by a single artery^3^^)^. If idiopathic sudden deafness is of vascular origin, it would be reasonable to expect this condition to have similar risk factors to other vascular diseases, such as coronary heart disease and stroke. However, there appear to be few epidemiological data about relationships between traditional cardiovascular risk factors and risks of idiopathic sudden deafness. We therefore conducted a nationwide case-control study in Japan to investigate whether known determinants of cardiovascular disease were associated with risks of idiopathic sudden deafness. An earlier report from this case-control study^4^^)^ showed that high intake of “Western” foods, and/or low intake of “Japanese” foods, were associated with increased risks of idiopathic sudden deafness. In this report, we aim to quantify the associations between smoking and alcohol intake and the risks of this condition. Associations with sleep duration were also investigated because of previous (albeit fairly weak) evidence that this might be a risk factor for idiopathic sudden deafness^5^^)^.

## METHODS

### Cases and Controls

Idiopathic sudden deafness in this study was diagnosed strictly according to criteria established by Japan’s Sudden Deafness Research Committee^6^^)^. The criteria specify sensorineural hearing loss of sudden onset, no involvement of cranial nerves other than the eighth nerve, and no known aetiology. The cases were consecutive patients diagnosed with idiopathic sudden deafness between October 1996 and August 1998 at collaborating hospitals, and who had an audiogram within 14 days of onset of this condition. All cases were recruited prospectively. Of the 171 eligible patients, 164 were included as cases in this study (4 patients were excluded as cases because no age-, gender- and residential district-matched controls could be obtained, and 3 patients were excluded because the otorhinolaryngologist (TN) who interpreted the audiograms could not identify which audiogram was for the ear affected by sudden deafness). The mean hearing loss in the affected and unaffected ears of cases has been reported elsewhere^4^^)^. For the purpose of subgroup analyses, the pattern of hearing loss in the cases was investigated by audiogram and classified into five subgroups using a modified version of Nakashima’s classification^4^^, ^^7^^)^.

The controls were obtained from a database of pooled controls that includes 73,861 people aged between 20 and 79 years and is held by the Research Committee on the Epidemiology of Intractable Diseases^8^^)^. The information in the database was obtained by questionnaire surveys conducted between 1987 and 1994. The total population of controls in this study is referred to hereafter as Controls I . A subgroup of these controls for whom the data collection period was closest (1993-94) to the data collection period for cases (1996-98) was also specified, and is referred to as Controls II . Controls I were matched to cases on age (in five-year bands), gender, and residential district (using the 12 districts defined in the annual National Nutrition Survey^9^^)^), whereas Controls II were matched only on age (also in five-year bands) and gender because of relatively sparse data. For each case, every available control in the population of pooled controls was selected^10^^, ^^11^^)^. Data were obtained for a total of 20,313 controls (Controls I ), of whom 2,964 were also Controls II . More detailed information about case and control selection has been reported elsewhere^4^^)^.

### Questionnaire

The exposure variables investigated in this paper were habitual tobacco smoking, alcohol intake and sleep duration. Information on these exposures, as well as information about potential confounders, was obtained using a self-administered questionnaire^12^^)^, which asked about exposure levels during the year before onset of sudden deafness (for cases) or during the year before completing the questionnaire (for controls). To ensure confidentiality, case questionnaires could be identified only using an identification number, and clinicians were not asked to check the responses. Cigarette smoking was assessed from eight questions about current and previous smoking habits, and participants were divided into three categories of smoking status: current smoker of 20 or more cigarettes per day, current smoker of fewer than 20 cigarettes per day, and non-smoker (which included past smokers). Habitual alcohol intake was assessed from seven questions about current and previous drinking habits, from which participants were divided into three categories: current habitual drinker of two or more units per day (one unit = approximately 27 g of ethanol); current habitual drinker of less than two units per day; and non-drinker (not a current habitual drinker). Sleep duration was assessed by a question about the average number of hours of sleep per 24 hours (referred to hereafter as hours of sleep per night). Participants were divided into three categories of sleep duration: less than 7 hours; greater than or equal to 7 hours but less than 8 hours; and greater than or equal to 8 hours. Information about dietary habit was obtained from a food frequency questionnaire^4^^)^.

### Statistical Methods

The statistical analyses were carried out using the SAS statistical package (version 6.12)^13^^)^. As is appropriate for the method of control selection in this study^4^^)^, the proportion of controls at each exposure level was directly standardised for age, gender and residential district in the case of Controls I , and for age and gender in the case of Controls II . Odds ratios (OR) and 95% confidence intervals (95% CI) were estimated using conditional logistic regression for group matching, where the matching variables were age, gender, and (where appropriate) residential district. All reported odds ratios were estimated from such models unless stated otherwise. For each exposure variable, additional adjustments were made for the other exposure variables in this paper (i.e. tobacco smoking, alcohol intake or sleep duration, as appropriate), and for dietary habit^4^^)^, by inclusion of these variables as covariates in the logistic models. Participants with one or more missing covariate values were not included in the regression analyses for analyses in which these covariates were specified. P-values for trend were estimated by conditional logistic regression using a continuous score assigned to each category of the exposure variables.

## RESULTS

### Characteristics of Study Participants

The characteristics of the cases and controls are shown in Table 1. Both Controls I and II were on average three years older than the cases. Roughly equal proportions of the cases and Controls II were men and women, but a larger proportion of Controls I was women (54.6%) than men (45.4%).

**Table 1.** Selected characteristics of cases and controls.

Characteristics		Cases (n=164)		Controls I (n=20,313)			Controls II (n=2,964)		
		
		Number/Total	%	Number/Total	%	%^a)^	Number/Total	%	%^b)^
Age, mean (SD), years		49.7 (14.1)		52.5 (10.1)			52.3 (12.2)		

Gender	men	83/164	50.6	9217/20313	45.4		1479/2964	49.9	

Cigarettes per day	0	105/154	68.2	10783/16043	67.2	70.5	2164/2952	73.3	72.5
	>0 and <20	12/154	7.8	1780/16043	11.1	9.0	249/2952	8.4	8.5
	≧ 20	37/154	24.0	3480/16043	21.7	20.5	539/2952	18.3	19.0

Alcohol intake, units per day^c)^	0	68/147	46.3	8346/13392	62.3	53.1	1437/2633	54.6	53.4
	>0 and <2	43/147	29.3	2581/13392	19.3	28.0	711/2633	27.0	28.1
	≧ 2	36/147	24.5	2465/13392	18.4	18.9	485/2633	18.4	18.3

Sleep duration, hours	<7	65/158	41.1	2224/9287	24.0	28.1	804/2929	27.5	28.8
	≧ 7 and <8	52/158	32.9	3363/9287	36.2	39.7	1152/2929	39.3	40.5
	≧ 8	41/158	26.0	3700/9287	39.8	32.3	973/2929	33.2	30.7

a) Adjusted for age, gender and residential district (see text).

b) Adjusted for age and gender (see text).

c) One unit contains about 27g of ethanol.

### Main Effect Estimates

Table 2 shows the odds ratios and 95% confidence intervals of sudden deafness for tobacco smoking, alcohol intake and sleep duration. There was extremely weak evidence of an association between daily tobacco intake and sudden deafness. Participants who smoked 20 or more cigarettes per day appeared to be about one-and-a-half times (OR=1.38, 95% CI=0.85-2.24 for Controls I , and OR=1.56, 95% CI=0.97-2.51 for Controls II ) as likely as non-smokers to have been cases. The p-values for trend across smoking categories were 0.22 (Controls I ) and 0.18 (Controls II ).

**Table 2.** Odds ratios of sudden deafness for cigarette smoking, alcohol intake and sleep duration.

Exposure variables		Controls I (n=20,313)						Controls II (n=2,964)					
	
					Multi-variable adjusted						Multi-variable adjusted		
	
		OR^a)^	95%CI	P-value for trend	OR^a,c)^	95%CI	P-value for trend	OR^b)^	95%CI	P-value for trend	OR^b,c)^	95%CI	P-value for trend
Cigarettes per day	0	1.00		0.219	1.00		0.388	1.00		0.179	1.00		0.321
	>0 and <20	0.90	0.47-1.72		0.81	0.41-1.63		1.03	0.54-1.97		1.14	0.53-2.49	
	≧ 20	1.38	0.85-2.24		1.28	0.77-2.13		1.56	0.97-2.51		1.35	0.75-2.44	

Alcohol intake, units per day^d)^	0	1.00		0.016	1.00		0.016	1.00		0.054	1.00		0.078
	>0 and <2	1.40	0.89-2.20		1.43	0.90-2.27		1.42	0.91-2.22		0.99	0.57-1.74	
	≧ 2	1.90	1.12-3.21		1.92	1.12-3.29		2.01	1.18-3.43		1.85	0.98-3.47	

Sleep duration, hours	<7	1.61	1.09-2.37	0.002	1.63	1.07-2.45	0.002	1.88	1.29-2.73	0.060	2.05	1.26-3.33	0.075
	≧ 7 and <8	1.00			1.00			1.00			1.00		
	≧ 8	0.98	0.63-1.52		1.01	0.63-1.63		1.00	0.65-1.55		1.27	0.73-2.22	

a) Matched for age, gender and residential district (see text).

b) Matched for age and gender (see text).

c) Adjusted for all other variables in the table and for dietary habit.

d) One unit contains about 27g of ethanol.

Participants who habitually consumed more than two units of alcohol (approximately 54g of ethanol) per day were about twice (OR=1.90, 95% CI=1.12-3.21 for Controls I , and OR=2.01, 95% CI=1.18-3.43 for Controls II ) as likely as non-drinkers to have been cases. Participants who were habitual drinkers but consumed less than two units per day appeared to be about one-and-a-half times (OR=1.40, 95% CI=0.89-2.20 for Controls I , and OR=1.42, 95% CI=0.91-2.22 for Controls II ) as likely as non-drinkers to have been cases, and although this increased risk was not statistically significant, it contributed to modest evidence of a positive trend in risk of sudden deafness across alcohol intake categories (p-values for trend =0.02 for Controls I , and 0.05 for Controls II ).

Participants who reported sleeping less than seven hours per night were about one-and-a-half to two (OR=1.61, 95% CI=1.09-2.37 for Controls I , and OR=1.88, 95% CI=1.29-2.73 for Controls II ) times more likely to have been cases than participants who reported sleeping seven to eight hours per night. There was evidence of a positive trend in risk of sudden deafness across sleep duration categories (p-values for trend = 0.002 for Controls I , and 0.06 for Controls II ). Additional adjustment of each of the above odds ratios for all of the other exposure variables in this paper and for dietary habit^4^^)^ did not materially alter the results.

### Subgroup Effect Estimates

Table 3 shows the odds ratios and 95% confidence intervals of sudden deafness for each exposure variable by sudden deafness subgroup using Controls I . Participants who habitually consumed more than two units of alcohol per day were about two-and-a-half times (OR=2.49, 95% CI=1.05-5.91) as likely as non-drinkers to have had flat-type hearing loss, and about five-and-a-half times as likely as non-drinkers to have had profound hearing loss (OR=5.55, 95% CI=0.99-31.23). In both groups, there was evidence of a positive trend in risk of sudden deafness across alcohol intake categories (p-values for trend = 0.04 for flat-type hearing loss, and 0.06 for profound hearing loss). With regard to smoking and sleep duration, there were borderline direct associations between these exposures and risks of sudden deafness among the participants with flat-type hearing loss. Participants who smoked 20 or more cigarettes per day were about twice (OR=1.96, 95% CI=0.91-4.23) as likely as non-smokers to have had this form of hearing loss. Participants who reported habitually sleeping less than seven hours per night were about twice (OR=1.81, 95% CI=0.96-3.38) as likely as those who reported sleeping seven to eight hours per night to have had flat-type hearing loss. Additional adjustment of each of the above subgroup odds ratios for all of the other exposure variables in this paper and for dietary habit^4^^)^ did not materially alter the results.

**Table 3.** Odds ratios of sudden deafness for the exposure variables, by sudden deafness subtype.

Exposure variables		Type of hearing loss (Audiometric configurations)									
		High-frequency		Low-frequency		Flat-type		Profound		Other	
				
		Cases (20)		Cases (31)		Cases (54)		Cases (20)		Cases (39)	
		Controls I (5,487)		Controls I (5,061)		Controls I (6,587)		Controls I (4,208)		Controls I (6,445)	
				
		OR^a)^	95% CI	OR^a)^	95% CI	OR^a)^	95% CI	OR^a)^	95% CI	OR^a)^	95% CI
Cigarettes per day	0	1.00		1.00		1.00		1.00		1.00	
	>0 and <20	0.84	0.10-7.46	0.66	0.17-2.50	0.73	0.21-2.50	2.18	0.37-12.90	1.00	0.28-3.57
	≧ 20	2.35	0.60-9.25	0.45	0.14-1.41	1.96	0.91-4.23	3.76	0.60-23.43	0.99	0.36-2.73

Alcohol intake, units per day^b)^	0	1.00		1.00		1.00		1.00		1.00	
	>0 and <2	1.64	0.46-5.81	1.20	0.46-3.10	1.51	0.69-3.29	1.73	0.39-7.64	1.16	0.47-2.88
	≧ 2	2.26	0.49-10.30	0.75	0.22-2.52	2.49	1.05-5.91	5.55	0.99-31.23	1.54	0.52-4.60

Sleep duration, hours	<7	0.75	0.23-2.48	1.59	0.66-3.84	1.81	0.96-3.38	3.16	0.82-12.16	1.47	0.66-3.27
	≧ 7 and <8	1.00		1.00		1.00		1.00		1.00	
	≧ 8	0.97	0.32-2.98	1.16	0.41-3.28	0.56	0.25-1.24	2.96	0.74-11.78	1.09	0.46-2.57

a) Matched for age, gender and residential district (see text).

b) One unit contains about 27g of ethanol.

## DISCUSSION

In this nationwide case-control study, increased risks of idiopathic sudden deafness were observed among participants who consumed two or more units of alcohol per day, and among participants who slept less than seven hours per night. The direct association with alcohol intake was particularly strong for the people with profound hearing loss.

The main strengths of this study were relatively rigorous diagnostic procedures, reasonable case representativeness, and the investigation of deafness subtypes. The main weaknesses were a small number of cases (suggesting that the reported associations might just be chance findings), and potential biases in the estimation of odds ratios. The most important potential biases were uncontrolled confounding (for example, by occupational status or other cardiovascular risk factors) and exposure measurement error. However, there are few reasons to suspect that the magnitude of any misreporting of the exposures would have differed between cases and controls, since, for example, most of the cases would not have had any prior knowledge of the hypotheses being investigated in this study, the possible relationship between lifestyle factors and idiopathic sudden deafness is not general knowledge, and the questionnaire was self-administered (so observer bias is less likely). Therefore exposure measurement error could be expected to have been mainly non-differential with respect to the outcome, thereby tending to bias the effect estimates toward the null.

There appears to be only one previous peer-reviewed epidemiological study^5^^)^ that investigated associations of idiopathic sudden deafness with smoking, alcohol intake and sleep duration. The study was a case-control study that reported a slightly (but statistically insignificant) increased risk of sudden deafness among participants who slept less than six hours per night. No associations with tobacco or alcohol intake were observed in the study. It is not certain why we observed an association with alcohol intake, whereas the earlier case-control study did not. However, the highest intake category in the earlier study (> 5 drinks per week) contained a smaller proportion of heavy drinkers than the highest intake category in the present study (≥2 units per day), thereby potentially diluting any genuine increased risks in that category in the earlier study. In addition, the previous study (109 cases and 109 controls) was smaller than the present study (164 cases, 20,313 controls in Controls I and 2,964 controls in Controls II ), so it would have had less statistical power to detect genuine associations should they have existed.

To our knowledge, there have been no previous reports on associations of the various subgroups of idiopathic sudden deafness with smoking, alcohol intake or sleep duration. Investigating associations with particular audiometric configurations could possibly provide insights into causal mechanisms^7^^)^, since the audiograms in idiopathic sudden deafness tend to be very heterogeneous. For example, they can show hearing loss across a wide range of frequencies, or hearing loss at just high or low frequencies. This heterogeneity might reflect a variety of causes operating at different levels of the auditory system. In this study, the association between alcohol intake and idiopathic sudden deafness was stronger for profound hearing loss than for flat-type hearing loss. Flat-type hearing loss and profound hearing loss are qualitatively similar (they both have a broad range of frequencies with hearing loss), but the degree of hearing loss is more severe in profound hearing loss. As the odds ratio for alcohol intake was higher in profound hearing loss than in flat-type hearing loss, this may indicate a form of dose-response relationship.

The hypotheses that tobacco smoking^14^^)^ and alcohol intake are risk factors for idiopathic sudden deafness are biologically plausible. For example, tobacco smoking might cause micro-circulatory and/or haemostatic abnormalities in the cochlea in the same way that it can cause such abnormalities in blood vessels of the heart^15^^)^ or brain^15^^, ^^16^^)^. In addition, chemical toxins such as carbon monoxide in tobacco smoke might potentially impair hearing by mechanisms similar to ototoxic drugs^2^^)^. Alcohol might affect blood coagulation^17^^)^, or it might cause vasospasm among blood vessels in the cochlea in the same way that it can cause vasospasm in brain^18^^)^. In addition, there is evidence that recent heavy alcohol consumption may trigger cerebral infarction^19^^)^. However, any effects of alcohol on hearing might vary according to the amount consumed, as is the case for ischemic heart disease^20^^)^ and cerebral infarction^21^^)^. For instance, there is good evidence that moderate alcohol intake protects against these conditions, whereas heavy intake may increase risks of these conditions^20^^, ^^21^^)^. Moreover, the effect of alcohol on coagulation and fibrinolytic factors is thought to vary according to the quantity of alcohol^17^^)^. The hypothesis that reduced sleep duration is a risk factor for sudden deafness is perhaps less biologically plausible, although at least one epidemiological study^22^^)^ reported a weak association between short sleep duration and risk of coronary heart disease.

Our findings suggest a need for future epidemiological research studies to investigate possible lifestyle determinants of idiopathic sudden deafness. Useful study designs at this stage would include ecological studies, case-crossover studies and case-control studies. Ecological studies could compare the incidence of sudden deafness in various countries with measurements of lifestyle factors in these countries, although control for confounding might be difficult. Case-crossover studies^23^^)^ could investigate the effects of transient exposures (such as acute alcohol intake) on sudden deafness, and case-control studies larger than the present study could potentially allow more precise subgroup analyses, thereby potentially providing greater insights into aetiological mechanisms. Further biomedical research into the potential aetiological mechanisms by which alcohol intake, tobacco smoking or sleep might cause sudden deafness would also seem warranted.
